# Editorial: Adipokines and hormone-dependent cancers

**DOI:** 10.3389/fendo.2023.1340171

**Published:** 2023-12-01

**Authors:** Sebastiano Andò, Bruno M. Simões

**Affiliations:** ^1^ Department of Pharmacy, Health and Nutritional Sciences, University of Calabria, Rende, Italy; ^2^ Centro Sanitario, University of Calabria, Rende, Italy; ^3^ Manchester Breast Centre, Division of Cancer Sciences, School of Medical Sciences, Faculty of Biology, Medicine and Health, University of Manchester, Manchester Academic Health Science Centre, Manchester, United Kingdom

**Keywords:** adipokines, cancer, obesity, adiponectin, IGF-1, leptin

Over the last two decades it has been progressively recognised the functional role of adipose tissue as an active and immunological organ, giving particular evidence to the role of adipokines in influencing the growth and progression of obesity-related cancer (1, 2). Among the different adipokines, leptin circulating levels are proportionally related to the development of adipose mass (2). Endocrine, paracrine and autocrine mechanisms of leptin action may sustain different malignancies working on their growth, progression and invasiveness (3). Leptin and its receptor are overexpressed in human breast cancer samples, wherein it has been reported that they contribute to promote cancer stem cell phenotype and to mediate tumour-stromal interaction sustaining invasive growth of breast cancer cells (4). Leptin shows a strong association with tumour grading, metastatic dissemination and poor prognosis (5). It acts as a strong amplifier of oestrogen signalling through a double mechanism: first, an upregulation of aromatase gene expression via AP-1 elements on its promoter, enhancing local oestrogen production; second, a direct trans-activation of ERα even in the absence of its ligand (6, 7). All this establishes leptin as a potential therapeutical target to improve clinical outcomes in breast cancer patients. However, not much is known about the link between levels of circulating leptin and breast cancer risk and prognosis. In the present Research Topic, Luo et al. highlight a paradox in postmenopausal Chinese women: high levels of leptin exhibit a superior overall survival (DFS) compared with those with low levels of leptin. Lennon et al. described how this apparent paradoxical effect is lost when BMI increased to morbid obesity (8). However, in the Chinese population relative few people are extremely obese and the authors suggested that a moderately increased BMI tended to be a protective factor in prognostic assessment of breast cancer (8). The study reported in the present Research Topic classifies 22% of 182 patients investigated with a BMI>28 as obese patients but it was not specified the incidence of patients with a BMI>30 which categorizes the condition of obesity in the Western countries. Indeed, the obesity rates range from 2,6% for Chinese to 34,9% for American population (9). Thus, the ethnicity of the patients investigated may influence the outcomes described. Moreover, many observations of the obesity paradox in cancer reflect methodological issues, including the “crudeness” of BMI as an obesity measure. For instance, BMI is a relatively crude measure of body adiposity and body composition and does not differentiate between lean mass and fat mass (8). In turn, body composition varies with sex and ethnicity, such that there aren’t currently specific age-gender-ethnicity indices to define obesity in a standardized manner (8). Hence, alternate measures of body composition or adipose tissue are warranted. Gonzales and colleagues addressed this issue and showed that the obesity paradox displayed in 175 patients with different types of cancers, on the basis of their BMI, disappeared when obesity was defined using fat mass index and lean mass index separately (10).

Tsankof and Tziomalos in the present Research Topic critically described the effect of adiponectin as a player of hormone-dependent cancers. Low levels of adiponectin appear to be associated with higher risk for breast, cervical, endometrial, ovarian, and prostate cancers (11–13). However, studies with cancer cells *in vitro* and in mouse xenograft models reveal that adiponectin effects on breast cancer cell growth and proliferation appear to be controversial. For instance, numerous *in vitro* evidences demonstrated the inhibitory action of adiponectin on breast carcinogenesis in ERα-negative breast cancer cells (1). Adiponectin exerts its effect mainly by the LKB1/AMPK pathway which inhibits signalling pathways involved in cell cycle initiation, cell growth and survival such as extracellular signalling regulated kinase (ERK 1/2), phosphatidil-inositol-3-kinase (PI3K/Protein kinase B or AKT), c-jun N-terminal kinases and signalling transducer and activation transcription 3 (STAT3) (14). However, ERα transactivation can occur through the recruitment of LKB1 as ERα co-activator, impairing its capability to activate AMPK controlling cellular energy balance, and promoting cell proliferation on ERα-positive breast cancer (11, 15, 16). In addition, in ERα-positive breast cancer cells and MDA-MB-231 cells ectopically expressing ERα, low concentration of adiponectin promoted IGF-1R phosphorylation, enhancing IGF-1/IGF-1R signalling (16). This demonstrates the existence of crosstalk between ERα and IGF-1 signalling upon adiponectin exposure. Thus, any attempt to employ adiponectin or adiponectin receptor agonist-like antitumor agents for tailored treatment of obese breast cancer patients should be carefully considered according to ERα expression profile.

Another important factor to be considered is the signalling between intra-tumoral adiponectin and its receptors AdipoR1 and AdipoR2. Commonly, AdipoR1 exhibits higher expression level in normal tissues with respect to AdipoR2. Adipocytes isolated from human breast cancer adipose tissue explants exhibited lower level of adiponectin and an increased expression of AdipoR1 (17). An increased AdipoR1/adiponectin ratio is also found in breast cancer cell lines treated with conditioned medium from human adipose tumoral tissue (17). Thus, the enhanced level of AdipoR1 has been interpreted as a compensatory mechanism to overcome the low secretion of adiponectin by peri-tumoural adipocytes. Moreover, it has been evidenced that the low secretion of adiponectin in adipose tissue adjacent to malignant breast cancer is concomitant to a reduced expression of adipogenesis-related genes, including HSL, HOXC8, HOXC9 and FABP4. In contrast, pro-inflammatory cytokines, like TNF-α, monocyte chemoattractant protein 1 (MCP-1) and IL-6, are up-regulated (18). The unbalanced secretion of these cytokines by dysfunctional adipocytes observed in obese subjects fuels the activation of different signalling pathways in breast cancer cells, leading to MAPK phosphorylation, responsible of increased cell proliferation, survival, growth and anti-apoptotic effects (19). All this generates a permissive tumour microenvironment, featured by a low-grade chronic inflammatory status, sustaining tumour proliferation. In the present Research Topic, the pan-cancer analysis performed by Chen et al. showed how AdipoR1 and R2 display a wide positive correlation across cancers and hence are potential novel drug targets. Moreover, their work revealed that amplification is the most common genetic alteration of AdipoR1 and R2 in cancer cells. The tumour with the highest frequency of AdipoR1 alterations is breast cancer while the tumour with the highest alteration of AdipoR2 is the ovarian epithelial cancer. Regarding the expression of these receptors in the tumour immune-microenvironment, their study showed that both AdipoR1 and AdipoR2 were positively associated with CD4+ T cells and negatively associated with NK in most cancers. Both receptors exhibited correlation with immune checkpoints in the majority of cancers, suggesting they could be used as predictors of immunotherapy. For example, CD274 encoding PDL-1 was observed significantly correlated with both receptors. To further elucidate the molecular mechanisms through which adiponectin may regulate cancer growth and progression we need appropriate *in vivo* models to study the effects of adipokine circulating levels and its tissue concentration together with its receptor expression in tumour and stromal cells.

Obesity association with cancer incidence is linked with metabolic changes, namely increased levels of insulin, IGF-1, IGFBP-3 and leptin and lower levels of adiponectin. A growing body of epidemiological data suggest that high levels of IGF-1 represent a risk factor for the development of breast, prostate, colon, and lung cancer. IGF-1 crosstalk with insulin, sex hormones and adipokines has been reported to synergistically function in this process. It is worth to mention that insulin inhibits AdipoR1 promoter via a repressive nuclear inhibitory protein element, antagonizing adiponectin signalling and favouring the development of insulin resistance (20). IGF-1R and insulin receptor (INSR) have a high degree of structural homology particularly in the tyrosine domain; they can form a heterodimer and signal through many common mediators but the two receptor signalling axes exhibit functional variance (21). In addition, the expression levels of IGF-1R and INSR are predictive of cancer outcomes. Experimentally, the modulation of IGF-1R activity affects the growth of many types of tumor cells. As a result of these findings, intensive efforts are being directed towards investigating the IGF pathway as both a diagnostic marker and a therapeutic target in cancer therapy (22). Correlation between obesity and prostate cancer risk has been reported especially in abdominal obesity with a linear relationship between increasing BMI and prostate cancer patients (23). Prostate cancer cells overexpress IGF-1R and INSR and periprostatic adipose tissue secretes a variety of inflammatory factors that creates a tumour microenvironment and promotes the development of prostate cancer (24–27). IGF-1R is also overexpressed in more than 50% of PDAC and its higher expression was associated with shorter overall survival and relapse in patients. Moreover, IGF1/IGF1R pathway activation promotes breast cancer by altering the expression of proliferation and survival genes through the Ras/Raf/MAPK and PI3K/Akt pathways (28–30). Similar to IGF-1, oestrogen can play a role in breast cancer initiation through the MAPK and PI3K/Akt signalling pathways, and oestrogen and IGF-1 signalling exhibit cross-talk (31). The role of IGF-1 in endocrine-related cancer has been highlighted in the present Research Topic by Zhong et al. focusing in the therapeutical strategy to target IGF signalling. The authors highlighted three main approaches used to target IGF signalling: i) IGF-1R monoclonal antibodies which function mainly by blocking ligand receptor action inducing the internalization/degradation of IGF-1R and downregulating IGF-1R/INSR hybrid receptor (32, 33); ii) IGF-1R tyrosine kinase inhibitors which act by competing for the binding site of the IGF-1R kinase domain to ATP; iii) IGF-1/-2 monoclonal antibodies blocking activation of IGF-1R, INSR-A, and their hybrid receptor without affecting INSR-B and insulin function (34). However, resistance to IGF-1R inhibitors may arise from the compensatory activation of RTK signalling. Therefore, combination therapies with RTK inhibitors have been suggested (35, 36). In tamoxifen-resistant breast cancer it has been shown that inhibition of IGF-1R signalling could restore sensitivity to endocrine therapy and combined IGF-1R/mTOR inhibition shows synergistic effects (37). In conclusion IGF-1 plays an important role in obesity-associated endocrine-related cancer and its targeting in combination with other therapies may provide better treatment alternatives for the clinical management of obese cancer patients.

In summary, the intricate interplay between adipokines, particularly leptin and adiponectin, and their receptors, as well as the involvement of insulin and IGF-1 in obesity-related cancer, underscores the complexity of the tumour microenvironment. Further research using advanced *in vivo* models is crucial to unravel the molecular mechanisms driving obesity-associated endocrine-related cancers and to develop targeted therapeutic interventions.

## Author contributions

SA: Writing – original draft, Writing – review & editing. BS: Writing – original draft, Writing – review & editing.
